# Integrative Bayesian variable selection with gene-based informative priors for genome-wide association studies

**DOI:** 10.1186/s12863-014-0130-7

**Published:** 2014-12-10

**Authors:** Xiaoshuai Zhang, Fuzhong Xue, Hong Liu, Dianwen Zhu, Bin Peng, Joseph L Wiemels, Xiaowei Yang

**Affiliations:** Bayessoft, Inc., 2221 Caravaggio Drive, Davis, CA 95618 USA; School of Public Health, Shandong University, Jinan, Shandong 250012 China; Shandong Provincial Institute of Dermatology and Venereology, Shandong Academy of Medical Science, Jinan, Shandong 250022 China; School of Public Health, Chongqing Medical University, Chongqing, 400016 China; Department of Epidemiology and Biostatistics, University of California San Francisco, San Francisco, CA 94158 USA

**Keywords:** Biomarker discovery, Bayesian hierarchical modeling, Gene-based biomarkers, Bayesian variable selection, Integrative biomarker identification

## Abstract

**Background:**

Genome-wide Association Studies (GWAS) are typically designed to identify phenotype-associated single nucleotide polymorphisms (SNPs) *individually* using univariate analysis methods. Though providing valuable insights into genetic risks of common diseases, the genetic variants identified by GWAS generally account for only a small proportion of the total heritability for complex diseases. To solve this “missing heritability” problem, we implemented a strategy called integrative Bayesian Variable Selection (iBVS), which is based on a hierarchical model that incorporates an informative prior by considering the gene interrelationship as a network. It was applied here to both simulated and real data sets.

**Results:**

Simulation studies indicated that the iBVS method was advantageous in its performance with highest AUC in both variable selection and outcome prediction, when compared to Stepwise and LASSO based strategies. In an analysis of a leprosy case–control study, iBVS selected 94 SNPs as predictors, while LASSO selected 100 SNPs. The Stepwise regression yielded a more parsimonious model with only 3 SNPs. The prediction results demonstrated that the iBVS method had comparable performance with that of LASSO, but better than Stepwise strategies.

**Conclusions:**

The proposed iBVS strategy is a novel and valid method for Genome-wide Association Studies, with the additional advantage in that it produces more interpretable posterior probabilities for each variable unlike LASSO and other penalized regression methods.

**Electronic supplementary material:**

The online version of this article (doi:10.1186/s12863-014-0130-7) contains supplementary material, which is available to authorized users.

## Background

Over the last decade, Genome-wide Association Studies (GWAS) have identified genetic loci associated for a variety of diseases [1-5]. Most studies aim to identify single nucleotide polymorphisms (SNPs) individually using univariate analysis methods [6]. Although current GWAS analyses have provided valuable insights into genetic risks of common diseases, the genetic variants identified by GWAS generally only account for a small proportion of the total heritability of complex diseases, illustrating a problem commonly referred to as “missing heritability” [7,8]. Potential explanations for this problem include the underestimation of the effects of alleles identified, the existence of gene-gene joint effects, the contribution of rare variation, the possibility that inherited epigenetic factors lead to correlated phenotypes between relatives, and the possible overestimation of heritability of the complex traits [7,9,10]. Many diseases or phenotypes are likely caused by or associated with multiple SNPs, each having small effects individually, but collectively contributing a more significant genetic effect [11]. Therefore, multi-locus SNP models would offer one appealing solution in capturing the underlying genotypic-phenotypic relationship [12-14].

A typical GWAS study measures thousands or millions of SNPs, but the number of subjects is usually much smaller. This is known as the *P* > > *N* problem [15,16]. One solution to this problem resorts to dimension reduction by identification of the optimal subset of predictors associated with the outcome variable. Determining the best model or selecting a subset of variables becomes an important statistical task for this method. Bayesian variable selection (BVS) provides a natural approach to solve this problem [17,18]. Unlike penalized regression approaches, BVS naturally produces easily interpretable measures of confidence for variable selection, *i.e*., posterior selection probabilities. This is an appealing advantage in GWAS because the primary goal of the analysis is to identify the joint effect of SNPs, and to utilize these identifications to learn about underlying biology. BVS has been successfully applied to GWAS data; see the discussion by Guan [19]. The flexibility of the BVS approach allows for straightforward extensions to analyze both quantitative and qualitative data [20-23]. Alternative techniques such as the single-SNP test, Stepwise regression, and LASSO (Least Absolute Shrinkage and Selection Operator) were developed to address this statistical challenge. LASSO is a regression method that involves penalizing the absolute size of the regression coefficients, and Stepwise is a classic scheme for sequentially adding to or removing variables from the model. Many studies show that BVS has better performance than these alternative methods in other contexts [19,23-25].

Diseases with complex inheritance may be influenced by multiple genes that interplay within genetic networks or pathways [26,27]. Gene products interact with one another and work collaboratively within interconnected pathways explaining or associating with certain diseases. This idea led to the concept of network-based molecular biomarkers. Stingo et al. [28] proposed a Bayesian modeling strategy that addressed this concept by incorporating biological information, which was based on the structure or topology of regulatory gene-gene networks in the analysis of DNA microarray data. The method was further generalized into a 2-step framework, STS (screening, then selection) by our research team [29] where standard methods were applied in the screening step to identify a set of candidate genes which were further explored in the selection step using the BVS strategy. In addition to these two coherent steps, our strategy involves the mapping of genotype data to gene-gene networks constructed from various sources such as protein-protein interaction networks [30,31]. We call this strategy of Bayesian biomarker discovery “integrated BVS” or iBVS. A partial least squares (PLS) g-prior for regression coefficients is also incorporated to solve the problem of non-positive deterministic covariance matrix when the sample size is smaller than the number of genes selected.

**Figure 1:**
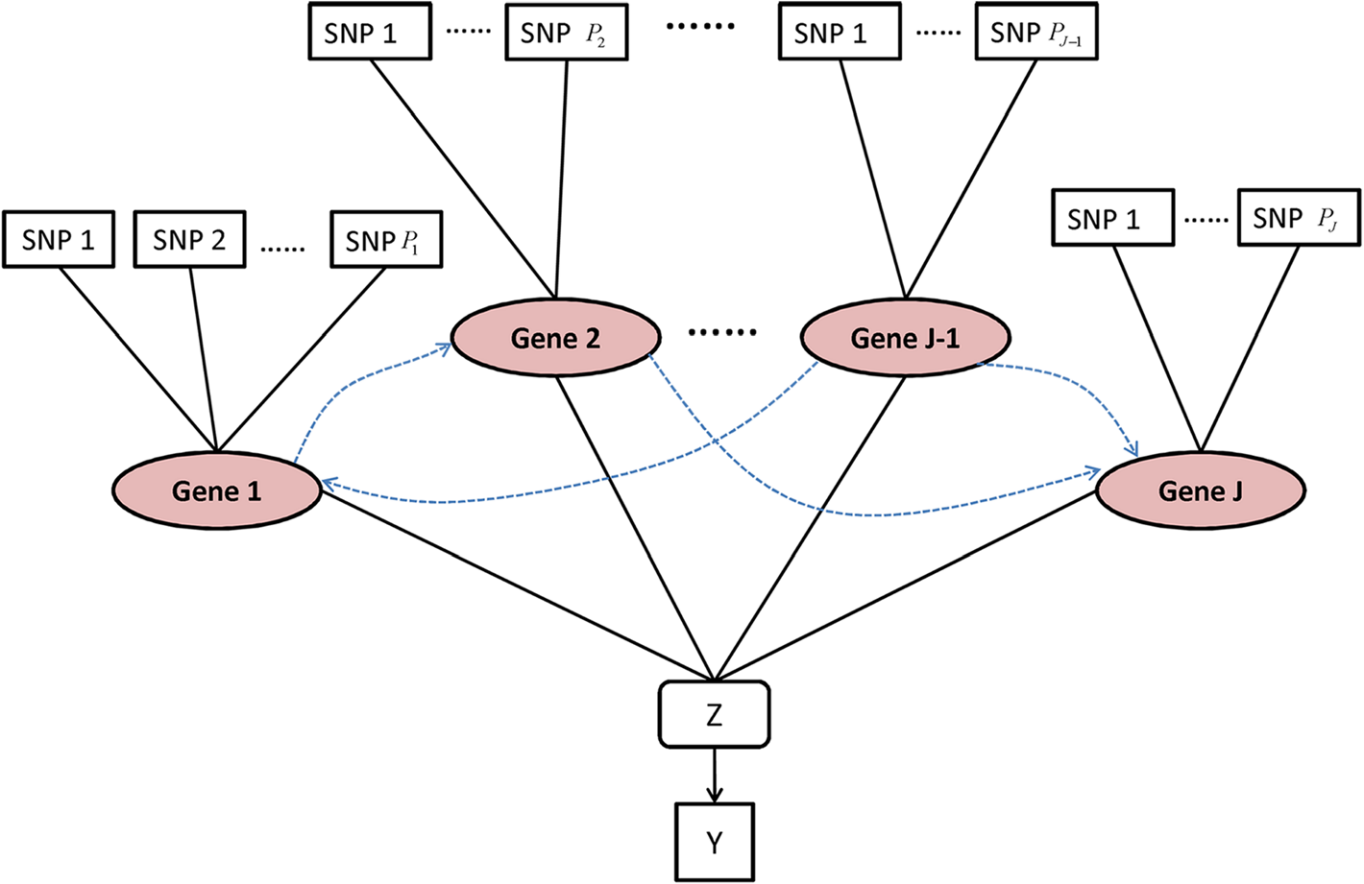
**Hierarchical model structure and relationships among the stochastic nodes.**

In this paper, we develop the strategy of iBVS for analyzing high dimensional GWAS data sets. The strategy is built upon a three-level hierarchical model as seen in Figure 1, where at the top level the PLS method is used to summarize the joint effect of selected SNPs within each gene. In the middle level, Markov Random Field (MRF) is employed to model the selection of genes in prediction of association with disease status. A focus of this article is on discovering SNPs within specific genes incorporating gene network information in GWAS under case–control design. Identification and prediction performance of this iBVS approach are then compared with those of LASSO and Stepwise selection strategies through simulation studies. We then apply iBVS to a GWAS data set for the prediction of leprosy, a skin disease, among Han Chinese.

## Methods

We denote *Y* = (*Y*_1_, ⋯ *Y*_*n*_) ' as independently observed binary outcomes in a GWAS data set, where *n* is the number of subjects and $$ {Y}_i=\left\{\begin{array}{c}\hfill 1\hfill \\ {}\hfill 0\hfill \end{array}\right.\begin{array}{c}\hfill \mathrm{if}\kern0.2em i\mathrm{t}\mathrm{h}\kern0.2em \mathrm{subject}\kern0.2em \mathrm{h}\mathrm{as}\ \mathrm{target}\ \mathrm{disease}\kern0.1em \hfill \\ {}\hfill \mathrm{otherwise}\hfill \end{array} $$. Each outcome is associated with a set of predictor variables, which correspond to the genotype data. We denote *x*_*ijk*_ as the genotype of the *k*^th^ SNP of the *j*^th^ gene on the *i*^th^ subject, for *i* = 1, ⋯, *n*, *j* = 1, ⋯, *J*, *k* = 1, ⋯, *P*_*j*_, where *P*_*j*_ is the number of SNPs mapped to the *j*^th^ gene, and $P={\displaystyle \sum_{j=1}^J{P}_j}$ denotes the total number of SNPs in the GWAS data set. Let *A* and *a* be the major and minor alleles at a SNP. The genotype may be coded according to different types of genetic effects: additive with 0, 1, 2 coding for the genotypes *AA*, *Aa*/*aA*, *aa*; dominant (with respect to the minor allele) with 0, 1, 1 coding for *AA*, *Aa*/*aA*, *aa*; Recessive (with respect to the minor allele) with 0, 0, 1 coding for *AA*, *Aa*/*aA*, *aa*.

### iBVS with hierarchical modeling for GWAS data

Figure 1 shows the proposed three-level hierarchical model structure. iBVS for binary phenotypes is accomplished by introducing the latent variable vector *Z* to the linear regression model. Each component *Z*_*i*_ ~ *N*(0, 1) is defined such that

$$ {Y}_i=\left\{\begin{array}{c}\hfill 0,\hfill \\ {}\hfill 1,\hfill \end{array}\right.\begin{array}{c}\hfill if\kern0.4em {Z}_i\le 0\hfill \\ {}\hfill if\kern0.3em {Z}_i>0\hfill \end{array}. $$

In order to select genetic variants in both gene and SNP level simultaneously, we introduce two binary vectors, *ξ* = (*ξ*_1_, ⋯, *ξ*_*J*_) and *γ* = (*γ*_1_, ⋯, *γ*_*P*_), to indicate the selection of genes and SNPs respectively into a model for predicting *Z*_*i*_, i. e,

$$ {\xi}_j=\left\{\begin{array}{c}\hfill 1\hfill \\ {}\hfill 0\hfill \end{array}\right.\begin{array}{c}\hfill if\kern0.2em j\mathrm{t}\mathrm{h}\kern0.2em \mathrm{gene}\kern0.2em \mathrm{is}\kern0.2em \mathrm{selected}\hfill \\ {}\hfill \mathrm{otherwise}\hfill \end{array}\left(j=1,\cdots, J\right) $$

and

$$ {\gamma}_p=\left\{\begin{array}{c}\hfill 1\hfill \\ {}\hfill 0\hfill \end{array}\right.\begin{array}{c}\hfill if\kern0.2em p\mathrm{t}\mathrm{h}\kern0.2em \mathrm{S}\mathrm{N}\mathrm{P}\kern0.2em \mathrm{is}\kern0.2em \mathrm{selected}\hfill \\ {}\hfill \mathrm{otherwise}\hfill \end{array}\left(p=1,\cdots, P\right). $$

For GWAS data with iBVS analysis, we propose the following hierarchical model,

$$ {Z}_i={\left({T}_{\left(\xi, \gamma \right)}{\beta}_{\left(\xi, \gamma \right)}\right)}_i+{\varepsilon}_i,{\varepsilon}_i\sim N\left(0,1\right), $$

where ${\beta}_{\left(\xi, \gamma \right)}=\left({\beta}_{J_1,\gamma },\cdots, {\beta}_{J_{\left|\xi \right|},\gamma}\right),{T}_{\left(\xi, \gamma \right)}=\left({T}_{\left({J}_1,\gamma \right)},\cdots, {T}_{\left({J}_{\left|\xi \right|},\gamma \right)}\right),\left|\xi \right|$ denotes the number of selected genes in predicting ${Z}_i,{T}_{\left({J}_l,\gamma \right)}$ denotes the vector of the first PLS component of ${X}_{J_l,\gamma },$ and *J*_*l*_(*l* = 1, ⋯, |*ξ*|) indexes the selected gene. Note that ${X}_{J_l,\gamma }$ is a sub-matrix of *X*, consisting of only the columns that correspond to selected SNPs in the selected genes.

### Prior specification

The indicator *γ* for SNP selection is assumed to follow an independent Bernoulli prior distributions with the same parameter *π* over all the *γ*_*i*_ values.

$$ p\left(\gamma \right)={\displaystyle \prod_{p=1}^P{\pi}^{\gamma_p}}{\left(1-\pi \right)}^{1-{\gamma}_p}0\le \pi \le 1 $$

Choice of *π* reflects a user’s prior belief in terms of the numbers of causal SNPs out of *P* candidates.

Zellner’s *g*-prior is commonly used for the regression coefficient parameters *β* [32]. Yang and Song [33] generalized the g-prior by modifying the matrix inverse to the Moore-Penrose generalized matrix inverse. Since the multicollinearity problem is commonly encountered in GWAS data because of strong linkage disequilibrium between SNPs, we took a similar prior as that of Yang and Song,

$$ {\beta}_{\left(\xi, \gamma \right)}\Big|\xi, \gamma \sim N\left(0,c{\left({T}_{\left(\xi, \gamma \right)}^{\prime }{T}_{\left(\xi, \gamma \right)}\right)}^{+}\right), $$

where ${\left({T}_{\left(\xi, \gamma \right)}^{\prime }{T}_{\left(\xi, \gamma \right)}\right)}^{+}$ denotes the Moore-Penrose generalized inverse of ${T}_{\left(\xi, \gamma \right)}^{\prime }{T}_{\left(\xi, \gamma \right)}$. This idea was first adopted by our research team for microarray gene expression data [29].

To take into account the pathway membership information for each gene as well as the biological relationships between genes within pathways, we follow Li and Zhang [34] and Stingo *et al*. [28] to use an MRF to describe the prior distribution on each component of the gene selection indicator, i.e.,

$$ p\left({\xi}_j\Big|{\xi}_i,i\in N{b}_j\right)\propto \exp \left({\xi}_j\left(\mu +\eta {\displaystyle \sum_{i\in N{b}_j}{\xi}_i}\right)\right) $$

where *μ* and *η* are tuning parameters, and *Nb*_*j*_ is the set of neighbors of gene *j* within the selected pathway. The corresponding multivariate form is given by:

$$ P\left(\xi \Big|\mu, \eta \right)\propto \exp \left(\mu {1}_J^{\prime}\xi +\eta {\xi}^{\prime }R\xi \right), $$

where ${1}_J^{\prime }$ is the vector consisting of *J* 1’s. We denote matrix *R* to reflect gene-gene network topological structure, where elements *R*_*ij*_ = 1 if there is a direct edge between the *i*^th^ and *j*^th^ genes, and *R*_*ij*_ = 0 otherwise.

### Posterior distributions

The joint posterior distribution of *θ* = (*γ*, *ξ*, *β*_(*ξ*,*γ*)_, *Z*) given (*Y*, *X*) is

$$ P\left(\gamma, \xi, {\beta}_{\left(\xi, \gamma \right)},Z\Big|Y,X\right)\propto \left({\displaystyle \prod_{i=1}^NI\left({A}_i\right)}\right)\times \exp \left[-\frac{{\left(Z-{T}_{\left(\xi, \gamma \right)}{\beta}_{\left(\xi, \gamma \right)}\right)}^{\prime}\left(Z-{T}_{\left(\xi, \gamma \right)}{\beta}_{\left(\xi, \gamma \right)}\right)}{2}\right]\times \exp \left[-\frac{\beta_{\left(\xi, \gamma \right)}^{\prime }{T}_{\left(\xi, \gamma \right)}^{\prime }{T}_{\left(\xi, \gamma \right)}{\beta}_{\left(\xi, \gamma \right)}}{2c}{\displaystyle \prod_{i=1}^{m_{\xi }}{\lambda}_i^{-\frac{1}{2}}}\right]\times {\displaystyle \prod_{p=1}^P{\pi}_p^{\gamma_p}}{\left(1-{\pi}_p\right)}^{1-{\gamma}_p}\times \exp \left(\mu {1}_J^{\prime}\xi +\eta {\xi}^{\prime }R\xi \right) $$

where *I*(*A*_*i*_) is the indicator function and *A*_*i*_ is either equal to {*Z*_*i*_ : *Z*_*i*_ > 0} or {*Z*_*i*_ : *Z*_*i*_ ≤ 0} corresponding to *Y*_*i*_ = 1 or *Y*_*i*_ = 0, respectively, and ${\lambda}_1,\cdots {\lambda}_{m_{\xi }}\left({m}_{\xi}\le J\right)$ are the nonzero eigenvalues of ${\left({T}_{\left(\xi, \gamma \right)}^{\prime }{T}_{\left(\xi, \gamma \right)}\right)}^{+}$.

Since *β* is a nuisance parameter, we can integrate it out to obtain the joint posterior distribution of (*Z*, *ξ*, *γ*|*Y*, *X*) as follows:

$$ P\left(Z,\xi, \gamma \Big|Y,X\right)\propto \left[{\displaystyle \prod_{i=1}^nI}\left({A}_i\right)\right]\times \frac{1}{{\left|{\varSigma}_{\left(\xi, \gamma \right)}\right|}^{1/2}} \exp \left(-\frac{Z^{\prime }{\varSigma}_{\left(\xi, \gamma \right)}^{-1}Z}{2}\right)\times {\displaystyle \prod_{p=1}^P{\pi_p}^{\gamma_p}}{\left(1-{\pi}_p\right)}^{1-{\gamma}_p}\times \exp \left(\mu {1}_J^{\prime}\xi +\eta {\xi}^{\prime }R\xi \right) $$

with ${\varSigma}_{\left(\xi, \gamma \right)}^{-1}=c{T}_{\left(\xi, \gamma \right)}{\left({T}_{\left(\xi, \gamma \right)}^{\prime }{T}_{\left(\xi, \gamma \right)}\right)}^{+}{T}_{\left(\xi, \gamma \right)}^{\prime }+{I}_n$. From this posterior joint distribution, we can derive the posterior conditional distributions

$$ P\left(Z\Big|\xi, \gamma, Y,T\right)\propto N\left(0,{\varSigma}_{\left(\xi, \gamma \right)}\right){\displaystyle \prod_{i=1}^nI}\left({A}_i\right), $$

which is a multivariate truncated normal distribution, and

$$ P\left(\xi, \gamma \Big|T,Z\right)\propto N\left(0,{\varSigma}_{\left(\xi, \gamma \right)}\right)\times {\displaystyle \prod_{p=1}^P{\pi_p}^{\gamma_p}}{\left(1-{\pi}_p\right)}^{1-{\gamma}_p}\times \exp \left(\mu {1}_J^{\prime}\xi +\eta {\xi}^{\prime }R\xi \right). $$

### Posterior inference via MCMC sampling

Markov chain Monte Carlo (MCMC) sampling is used to generate samples for the posterior distribution of the model parameters. The MCMC sampling procedure consists of the following two steps:

I. Sample *ξ* and *γ* from *P*(*ξ*, *γ*|*Y*, *T*, *Z*): the selection parameters (*ξ*, *γ*) are updated using a Metropolis-Hastings algorithm which is modified from Stingo’s method [28] (details of the MCMC moves to update (*ξ*, *γ*) are given in Additional file 1). The method consists of randomly picking one of the following moves: (1) change the inclusion status of SNP and gene by randomly choosing from adding or removing a gene and a SNP at the same time; (2) change the inclusion status of SNP only by randomly choosing from adding or removing a SNP.
II. Sample Z from *P*(*Z*|*Y*, *T*, *γ*, *ξ*): directly sampling from this distribution is known to be difficult. In this article, we follow the method given in Devroye [35] to simulate samples from the univariate truncated normal distribution *P*(*Z*_*i*_|*Z*_− *i*_, *Y*, *T*, *γ*, *ξ*), where *Z*_− *i*_ is the vector of Z without the *i*^th^ element.

### Simulation

Simulation studies were conducted to assess the performances of iBVS, LASSO regression, and Stepwise regression using a proprietary set of MatLab codes, an R package glmnet, and the R package lars. We simulated three scenarios of varying different proportion of variance of Z explained by the genetic factors, labeled as follows: (1) H70: genes with network, 70% explained variance; (2) H50: genes with network, 50% explained variance; and (3) H30: genes with network, 30% explained variance.

For each scenario, 50 sets of genotypes without disease status were simulated using software Hapgen2 [36] based on the genotype data from Hapmap project (http://hapmap.ncbi.nlm.nih.gov/). We subsequently generated phenotypes corresponding to each scenario, with 400 individuals and 300 SNPs assorted into 22 genes for each data set. The first 200 individuals were used to fit the iBVS hierarchical model and to evaluate the performance of identifying causal SNPs of the three methods, while the other 200 individuals were used to assess the prediction performance of each method. All the simulations were run under the additive and dominant genetic model respectively to check the model flexibility of the proposed iBVS.

We simulated sets of phenotypes in the following way: First, we specified 10 SNPs *x*_*j*_(j = 1 … 10) as causal SNPs, which were positioned within 5 genes. In order to add network information to gene levels, a network was simulated between the genes. We then conducted a precision matrix *Ω* which contains the network relationship between the genes. If there is an edge between *p*^th^ gene and *q*^th^ gene in the network, *ω*_*pq*_ and *ω*_*qp*_ would be assigned with a non-zero value, otherwise 0. The vector *t*_*i*_ was generated from a multivariate normal distribution with zero mean vector and covariance matrix *Σ* = *Ω*^− 1^. Then the causal gene score *T*_*k*_ (k = 1…5) was calculated by considering both genotype data and gene network information, ${T}_{ik}={\displaystyle \sum_{SNP\kern0.1em j\in gene\kern0.2em k}{b}_j{x}_{kj}+}\kern0.1em {t}_{ik},$ where *b*_*j*_’s were carefully chosen to indicate different PLS configurations. We subsequently simulated the latent phenotypes score for each individual using *z*_*i*_ = ∑*ϕ*_*j*_*T*_*ik*_ + *ε*_*i*_, *ε*_*i*_ ~ *N*(0, 1). Finally, binary phenotypes *Y*_*i*_ for each individual was generated using $$ {Y}_i=\left\{\begin{array}{c}\hfill 1\hfill \\ {}\hfill 0\hfill \end{array}\right.\begin{array}{c}\hfill \mathrm{if}\kern0.2em {z}_i\ge 0\kern0.1em \hfill \\ {}\hfill \mathrm{if}\ {z}_i<0\hfill \end{array} $$.

### Application

We applied iBVS, LASSO and Stepwise approaches to analyze a GWAS data set designed to identify genetic variants associated with leprosy [37]. The genotype data consisted of 492,109 SNPs from 706 cases and 514 controls after removing genetically unmatched controls, to obviate the need for correction for population stratification. All subjects were Han Chinese from eastern China. In order to select variables and assess the performance of the three variable selection strategies, we randomly divided the data set into two parts: a training set and a testing set, each with 610 samples. The training set was used for SNP selection, while the testing set for validating the selection results and comparing the three methods. The genotype was first coded under the additive genetic model, and we re-coded the genotypes following dominant genetic components and re-analyzed this real data set to check the model flexibility under different genetic effects.

## Results

### Simulation studies

#### Performance of variable selection

The average area under the curve (AUC) was calculated to evaluate the performance of casual SNP identification in each scenario. For SSVS, the AUC is calculated using the following formula [38,39]. $AUC=\frac{1}{n^D{n}^C}{\displaystyle \sum_{i\in D,j\in C}I}\left\{{\gamma}_i>{\gamma}_j\right\},$ where *D* is the set of the causal SNPs and *C* is the set of the non-causal SNPs; *n*^*D*^ and *n*^*C*^ are the number of causal and non-causal SNPs, respectively.

For the LASSO method, a simple modification of the Least Angle Regression (LAR) algorithm was implemented that calculates the entire LASSO path, which is an efficient way of computing the solution to any LASSO problem especially when *P* ≫ *N* [40]. Using the modified LARS algorithm, one may generate all LASSO solutions corresponding to different values of the penalty parameter. Selecting the active model at a given iteration would give one LASSO solution corresponding to a particular value of the penalty parameter. Hence, one can control the penalty parameter using a cutoff for the number of iterations [38]. For LASSO and Stepwise approaches, the following formula was used to calculate the AUC: $AUC=\frac{1}{n^D{n}^C}{\displaystyle \sum_{i\in D,j\in C}I}\left\{{s}_i<{s}_j\right\},$ where *s* is the iteration at which the *i*^th^ marker enters the model [38].

Table 1 shows the averaged AUC of the three methods in variable selection under the additive genetic model. It can be seen that the AUC of the three methods all increase monotonically by the proportion of variance of *Z* explained by the genetic factors. Obviously, under scenario H70 and H50, the iBVS has the highest averaged AUC (0.911 and 0.894) followed by Lasso (0.891 and 0.882), while the AUC of Stepwise is relative low (0.869 and 0.853). The AUC of iBVS drops to 0.792 with low explained variance (H30), with LASSO and Stepwise both approximating 0.77. The results revealed that iBVS has superior performance compared with that of LASSO and Stepwise regression. Similar trends can also be found under the dominant genetic model (Additional file 1: Table S2).

**Table 1:** **Average AUC values of iBVS, LASSO and Stepwise**

**Scenario**	**Average AUC**		
	**iBVS**	**LASSO**	**Stepwise**
**H70**	0.911	0.891	0.869
**H50**	0.894	0.882	0.853
**H30**	0.792	0.779	0.774

#### Performance of outcome prediction

We subsequently assessed the prediction performance of the three methods using the remaining 200 individuals. Prediction performances were evaluated by considering correctly/incorrectly predicted positive/negative outcomes. We calculated specificity and sensitivity for thresholds from 0.01 to 0.99, with steps of 0.01. Then the ROC was plotted using mean specificity and sensitivity for a given threshold. For iBVS, we use a two-stage strategy. First the posterior probabilities of all the SNPs were estimated by iBVS. The top *i* SNPs (*i* = 1…300) were subsequently fitted into a PLS logistic regression model, and 10-fold cross-validation was conducted to choose the optimal number of predictors *i*. For LASSO and Stepwise approaches, the optimal model was determined by a 10-fold cross-validation.

**Figure 2:**
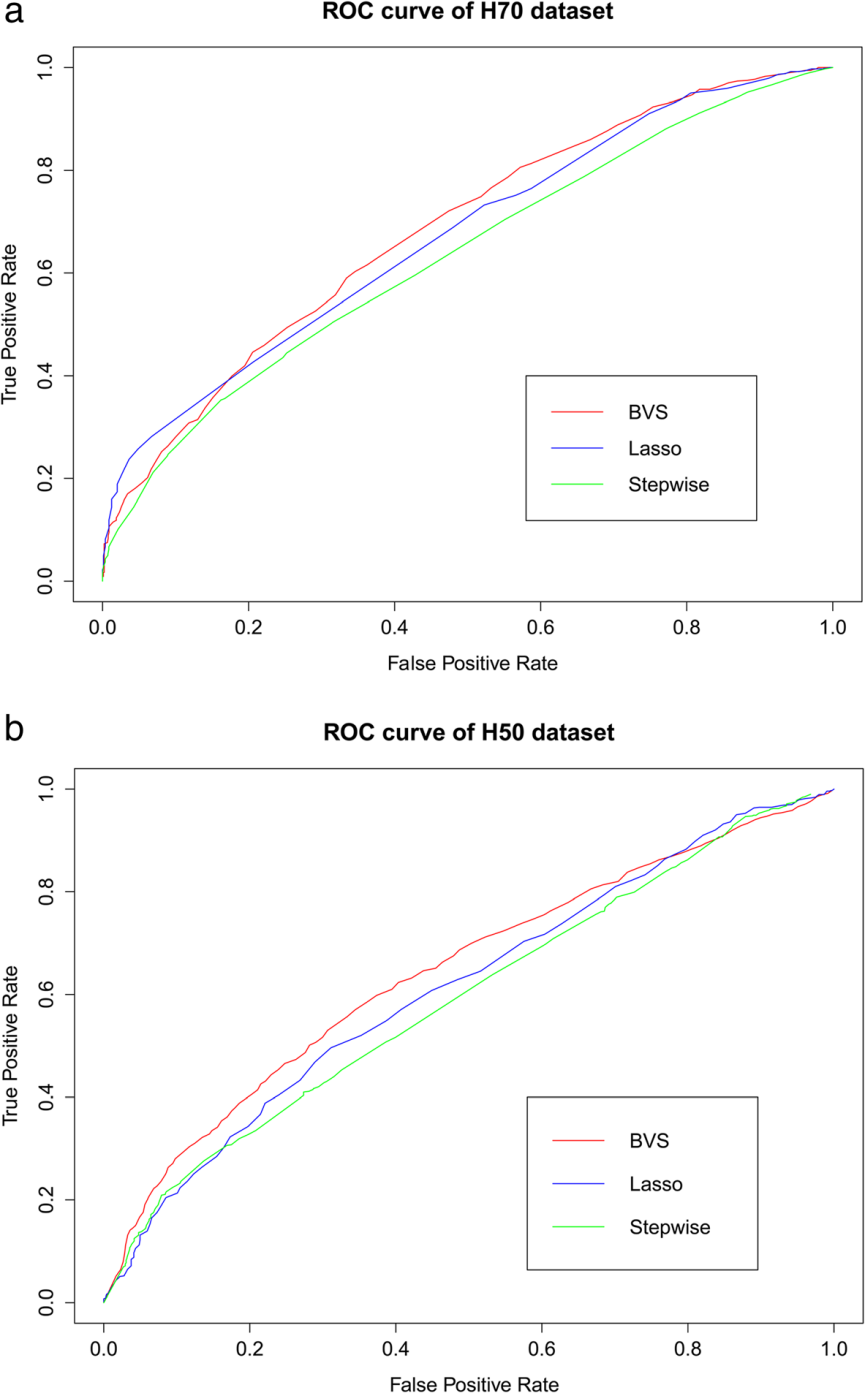
**ROC curves of the three SNP selection strategies on the simulated data.** Figure **(a)** depicts the ROC curves of the simulated data in scenario H70, and Figure **(b)** depicts the ROC curves of the simulated data in scenario H50.

Figure 2 demonstrates the ROC of the three methods in scenarios H70 and H50. One can see that the prediction performance of iBVS was slightly greater than that of LASSO, and had an obvious superiority to Stepwise regression.

### Application

We first conducted screening of all SNPs, one by one by fitting the single-SNP logistic regression model with additive coding. By sorting on the *p*-values from the univariate analysis, we identified 100 genes, each containing at least one SNP with P-value smaller than 0.0004. We subsequently extracted all of the SNPs in each significant gene to comprise the joint effect of SNPs per one gene. The 100 genes selected above contained a total of 3,388 SNPs.

**Figure 3:**
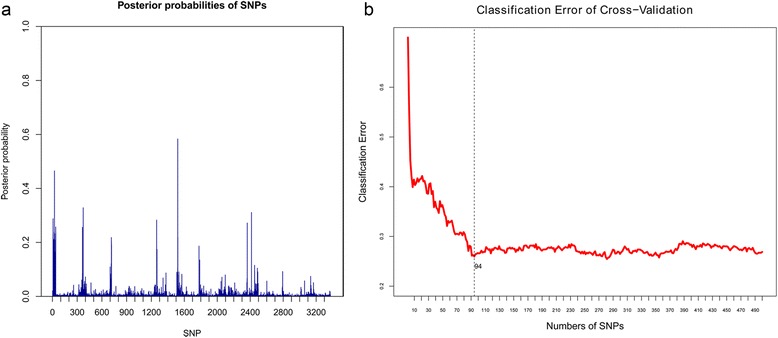
**Posterior selection probabilities of SNPs and result of cross-validation in leprosy training Data.** Figure **(a)** depicts the posterior SNP selection probabilities for the 3388 SNPs from the leprosy training data set. Figure **(b)** depicts the classification error in the conduct of cross-validation on the training data set using the PLS logistic regression model with different selection of top SNPs.

iBVS was applied using the above 3,388 SNPs. First, we constructed the *R* matrix using the KEGG database (details please see the Additional file 1). We subsequently specified prior distributions as described in the Methods Section, with hyper parameters set as: *π* = 0.01, *μ* = − 2, *η* = 0.8. Finally, the MCMC was conducted to make posterior inferences with 10,000 iterations as burn-in and 50,000 additional runs. Figure 3a shows the posterior SNP selection probabilities via our iBVS strategy with integrated biological priors.

A ten-fold cross-validation approach was employed to set a cut-off for determining the optimal prediction model. The top *i* SNPs were added into a PLS logistic model one by one, to estimate the cross-validation error, shown in Figure 3b. It can be seen that the smallest classification error appeared when the top 94 SNPs were selected as predictors. The classification error stabilizes after 94 SNPs were selected into the PLS logistic model, with some slight increase shown. Therefore the top 94 SNPs were selected as ‘significant’ predictors whose information was listed in (see the Additional file 1: Table S1).

**Figure 4:**
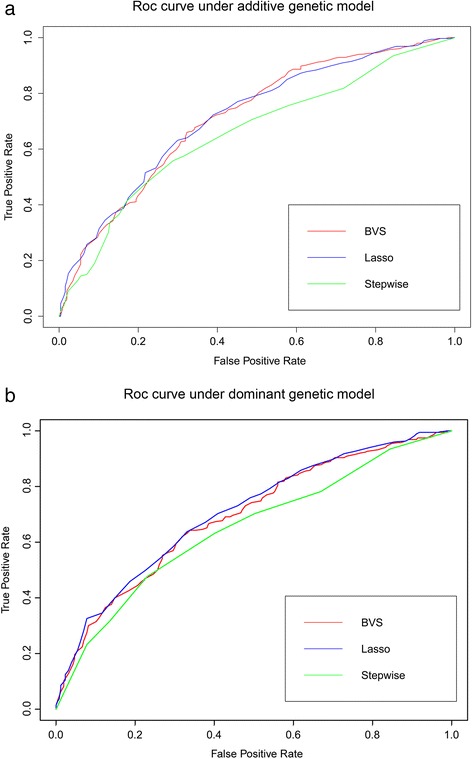
**ROC curves from leprosy testing data set under additive and dominant genetic model.** Figure **(a)** depicts the ROC curves of testing data set with different SNP selection strategies (iBVS, LASSO, and Stepwise) under additive genetic model, and Figure **(b)** under dominant genetic model.

In addition, we ran LASSO and Stepwise regression on this leprosy GWAS dataset, with the optimal model determined by 10-fold cross-validation. The LASSO selected 100 SNPs, which only included 24 SNPs selected by iBVS. The Stepwise regression approach yielded a more parsimonious model with only 3 SNPs. Table 2 shows the detailed information of 24 common SNPs selected by both iBVS and Lasso. Specifically, the three SNPs selected by Stepwise also had high corresponding posterior probability in iBVS and relative large coefficient in LASSO, and SNP rs9270984 is most significant in all three methods. Finally, we assessed the ability of the three methods to correctly predict binary responses (case versus control) of the test data set. Figure 4a shows the ROC curves of the three selected models under the additive genetic model, while Figure 4b demonstrates that under the dominant model. This indicates that iBVS has comparable performance to the LASSO model, but has an performance advantage over the Stepwise regression method no matter what the genetic model is.

**Table 2:** **Information of 24 common SNPs selected by both iBVS and Lasso**

**SNP**	**Chromosome**	**Position**	**Gene**	**Posterior probability**	**Lasso coefficient**	**Stepwise coefficient**
rs9270984	6	32681969	HLA-DR–DQ	0.583	0.307	0.195
rs7595482	2	38106517	FAM82A1	0.329	−0.031	-
rs10133203	14	51425137	GNG2	0.311	−0.314	-
rs2517467	6	30997239	VARS2	0.283	0.237	0.148
rs3764147	13	43355925	C13orf31	0.272	0.227	0.114
rs1446297	2	38061737	FAM82A1	0.256	−0.208	-
rs2237585	7	94887754	PON2	0.187	−0.222	-
rs42490	8	90847650	RIPK2	0.135	−0.143	-
rs602875	6	32681607	HLA-DR–DQ	0.104	−0.090	-
rs16945848	15	60913837	TLN2	0.093	0.245	-
rs241409	6	32969898	LOC100294145	0.082	0.018	-
rs12817755	12	38585079	SLC2A13	0.08	−0.137	-
rs1343104	20	57607136	PHACTR3	0.075	−0.06	-
rs10502281	11	123261833	TMEM225	0.071	−0.105	-
rs2305100	13	43346934	CCDC122	0.066	0.001	-
rs447833	20	42696770	ADA	0.058	0.209	-
rs11632705	15	25141046	GABRG3	0.057	−0.043	-
rs17065164	13	43342706	CCDC122	0.051	−0.066	-
rs11900859	2	138039737	THSD7B	0.051	0.071	-
rs241443	6	32905093	TAP2	0.045	0.18	-
rs1897419	2	137473187	THSD7B	0.045	0.023	-
rs1805867	8	91100250	DECR1	0.043	−0.126	-
rs2517598	6	30188253	TRIM31	0.043	0.248	-
rs17110817	14	80120188	CEP128	0.04	0.005	-

## Discussion

GWAS analyses typically approach data as a list of single SNPs, a strategy which has yielded a catalog of susceptibility loci for complex diseases. However, the statistically significant variants detected so far account for only a small proportion of the total phenotypic variation. Gene-based tests for association are increasingly being seen as useful complements to GWASs, demonstrating several attractive features compared with traditional SNP-based analysis [12-14]. Beyond gene-based methods, there is an increasing recognition of the potential contributions of pathway-based analyses, in which variants in groups of genes within specific pathways are considered together to predict the phenotype [41-43]. In this paper, we followed an integrative biomarker identification scheme to conduct a novel hierarchical model incorporating a gene-gene network or pathway information for SNP identification in GWAS via the Bayesian inference paradigm.

Three scenarios of data sets were simulated, each considering different proportions of variance of outcome explained by genetic factors. Simulation results show our iBVS method outperformed the LASSO and Stepwise methods in identifying causal SNPs in each scenario (Table 1). In addition, we also evaluate the prediction ability of the three methods using additional data sets, and show that iBVS had advantageous performance (Figure 2). The advantages of the proposed iBVS strategy is verified when the real network is known and explicitly employed through a prior specification with the MRF distribution.

After applying iBVS to an actual GWAS dataset, a panel of 94 SNPs were selected as predictors of leprosy. The LASSO method selected 100 SNPs, which included only 24 SNPs in common with iBVS. The Stepwise regression yielded a very parsimonious model with only 3 SNPs. The results indicate that the iBVS method has comparable prediction performance with LASSO, and advantageous with Stepwise. Moreover, all the results are quite stable under the different genetic models. Stepwise regression searches the model space by adding or removing one SNP at a time and therefore the searching is partial, leading to convergence at a local optimum. The reason iBVS does not outperform LASSO may be due to deficiencies in pathway information from existing databases that do not reflect complete signaling pathways. The performance of iBVS can be improved by developing a stochastic inference of the gene-gene networks from the data and merging it into the current iBVS MCMC algorithm, which remains a future goal. Compared with LASSO and other penalized regression methods, which lack appropriate interpretation, iBVS has an additional advantage in that it produces posterior probabilities for each variable. This is a particularly important advantage in GWAS because the primary goal of the analysis is to identify the effect of SNPs. Comparing to metrics like *p*-values, posterior probabilities have clear interpretation. A reviewer of this article suggested that many important traits are generally quantitative and are often controlled by multiple genes in shared biology pathways; our model could be naturally extended to analyze quantitative data by removing the latent variable *Z* from the hierarchical model.

Efficiency of stochastic algorithms often diminishes as the total number of variables increases [19,21]. It would be appealing to remove noisy data points or those with lower quality before using a stochastic search. Therefore, we first screened the total number of SNPs using a conventional SNP-based model to filter the number of SNPs included in the Bayesian hierarchical model. This set of candidate SNPs and their associated genes is called the ‘signature set’ in the sense that they are possibly signaling SNPs/genes (in other words, causal or marker SNPs/genes). We extracted all the SNPs in each significant gene to comprise the joint effect of a gene, leaving the weaker candidate genes out of the iBVS. The screening step should be viewed as a general step not only for dimension reduction but for constructing a functional context before conducting BVS.

A few issues regarding our model choices and computation should be highlighted. We mainly adopted the perspective of subjective Bayesian analysis due to the fact that we want to incorporate informative priors from relevant scientific sources. Choosing an objective prior that satisfies some fundamental principles as summarized in Bayarri et al. [44] would be theoretically appealing in future work. Another issue concerns computational burden. With a large number of parameters in the model, the inference is mainly based on Monte Carlo simulation, which may take a prolonged time. Running over a single computer with 3.3GH CPU computer and 8GB memory, 6 days were required to finish the leprosy data analysis. With the advent of high-speed cluster computers and the existence of cloud computing technologies, it is becoming increasingly feasible to apply full iBVS methods for biomarker identification.

## Conclusions

We proposed an iBVS method to analyze high dimensional GWAS data sets based on a hierarchical model that incorporates an informative prior on networked gene interrelationships. Simulation studies showed that our iBVS method had better performance in both biomarker identification and disease prediction than LASSO and Stepwise models. A leprosy GWAS analysis showed iBVS method demonstrated a comparable performance with LASSO, and better than Stepwise methods. iBVS did not outperform LASSO, which may be due to deficiencies in existing signaling pathway databases that are likely to be improved as the knowledge base increases. In summary, the proposed iBVS strategy is a valid method for GWAS, having an additional advantage in the production of posterior probabilities for each variable that are again subject to continued refinement.

## Additional file

Additional file 1:
**MCMC Scheme for Sampling (** $\boldsymbol{\upxi}$ ***, y*** **).** **Table S1.** Information of the selected 94 SNPs. **Table S2.** Additional simulation results under dominant genetic model. **Figure S1.** Pairwise correlation coefficient R square of the 94 top SNPs. **Figure S2.** Posterior selection probabilities of SNPs under dominant genetic model in leprosy GWAS analysis. **Constructing R Matrix Using KEGG**.

## Abbreviations

iBVS: Integrative Bayesian variable selection
LASSO: Least absolute shrinkage and selection operator
STS: Screening, then selection
AUC: The average area under the curve
