# Supplementary material for: Integrative Bayesian variable selection with gene-based informative priors for genome-wide association studies
Source: BMC Genet. 2014 Dec 10;15:130. doi: 10.1186/s12863-014-0130-7 (PMC4275962; doi:10.1186/s12863-014-0130-7)
Supplement: Additional file 1: — MCMC Scheme for Sampling ( $\boldsymbol{\upxi}$ξ , y ). Table S1. Information of the selected 94 SNPs. Table S2. Additional simulation results under dominant genetic model. Figure S1. Pairwise correlation coefficient R square of the 94 top SNPs. Figure S2. Posterior selection probabilities of SNPs under dominant genetic model in leprosy GWAS analysis. Constructing R Matrix Using KEGG. [file 12863_2014_130_MOESM1_ESM.pdf]

MCMC Scheme for Sampling (ξ, γ)

Here we aim to describe the MCMC steps for (ξ, γ) in more detail. At each iteration, only one gene and/or a SNP are proposed to be added or removed.

(1) Change inclusion status of both gene and SNP – randomly choose between addition (move 1a) or removal (move 1b):

(1a) Add a gene and a SNP: First select a gene that is not included in the model and has none of its SNPs in the model ($\xi_k^o = 0$ and $p_{k\gamma}^o = 0$). Randomly choose one SNP from the gene ($\gamma_j^o = 0$) and propose including both the gene and the SNP, i.e., set $\xi_k^p = 1$, $\gamma_j^p = 1$. The move is accepted with probability

$$\min\left\{1, \frac{f(\xi^p, \gamma^p \mid T, Z)}{f(\xi^o, \gamma^o \mid T, Z)} \cdot \frac{p_k \cdot \sum_{r=1}^J I\{\xi_r^o = 0, p_r^o = 0\}}{\sum_{r=1}^J I\{\xi_r^p = 1, p_{r\gamma}^p = 1\}}\right\}$$

(1b) Remove a gene and a SNP:

This move is the reverse of (1a) described above. We first select a gene that is included in the model and has only one of its member SNPs in the model ($\xi_k^o = 1$ and $p_{k\gamma}^o = 1$). We attempt to remove both the pathway and the gene, i.e., set $\xi_k^o = 1$, $\gamma_j^o = 1$ and accept the move with probability

$$\min\left\{1, \frac{f(\xi^p, \gamma^p \mid T, Z)}{f(\xi^o, \gamma^o \mid T, Z)} \cdot \frac{\sum_{r=1}^J I\{\xi_r^o = 1, p_r^o = 1\}}{p_k \cdot \sum_{r=1}^J I\{\xi_r^p = 0, p_r^p = 0\}}\right\}$$

(2) Change the inclusion status of SNP but not gene – randomly choose between addition (2a) or removal (2b):

(2a) Add a SNP in an already included gene: First select a gene that has already been included in the model and has some member SNPs that could potentially be added

($\xi_k^o = 1$ and $p_k > p_k^o$). Let G be the set of genes that satisfy this criterion. Choose one of the non-included SNPs from this gene and attempt to add it, i.e, set $\xi_k^p = \xi_k^o = 1, \gamma_j^p = 1$. The proposal is accepted with probability

$$\min\left\{1, \frac{f(\xi^p, \gamma^p \mid T, Z)}{f(\xi^o, \gamma^o \mid T, Z)} \cdot \frac{\sum_{r=1}^{J} I\{\xi_r^o = 1, p_k > p_{r\gamma}^o 1\} \cdot \sum_{r \in G} \frac{1}{p_{r\gamma}^p}}{\sum_{r=1}^{J} I\{\xi_r^p = 1, p_{r\gamma}^p > 1\} \cdot \sum_{r \in G} \frac{1}{p_r - p_{r\gamma}^o}}\right\}$$

(2b) Remove a SNP from an already included gene:

This move is the reverse of (2a) described above. We first select a gene that has been already included in the model and that has more than one of its member SNPs included in the model ($\xi_k^o = 1$ and $p_{k\gamma}^o > 1$). Once the gene is selected, choose a SNP among the eligible candidates, that is, an included member SNP ($\gamma_j^o = 1$). Leave the gene status unchanged and attempt to remove the selected SNP, i.e., set $\xi_k^p = \xi_k^o = 1, \gamma_j^p = 0$. The proposed move is accepted with probability

$$\min\left\{1, \frac{f(\xi^p, \gamma^p \mid T, Z)}{f(\xi^o, \gamma^o \mid T, Z)} \cdot \frac{\sum_{r=1}^{J} I\{\xi_r^p = 1, p_{r\gamma}^p > 1\} \cdot \sum_{r \in G} \frac{1}{p_r - p_{r\gamma}^o}}{\sum_{r=1}^{J} I\{\xi_r^o = 1, p_k > p_{r\gamma}^o 1\} \cdot \sum_{r \in G} \frac{1}{p_{r\gamma}^p}}\right\}$$

Table S1. Information of the selected 94 SNPs

No	SNP	Chromosome	Position	Gene	Posterior Probability	effect
1	rs9270984	6	32681969	HLA-DR–DQ	0.5835	0.4097
2	rs501080	1	18907980	PAX7	0.4653	0.0564
3	rs7595482	2	38106517	FAM82A1	0.3287	-0.2253
4	rs10133203	14	51425137	GNG2	0.3112	-0.3338
5	rs6695765	1	18851907	PAX7	0.2878	-0.0695
6	rs2517467	6	30997239	VARS2	0.2831	0.2671
7	rs3764147	13	43355925	C13orf31	0.2721	0.2741
8	rs9438551	1	26647810	DHDDS	0.2573	-0.1279
9	rs1446297	2	38061737	FAM82A1	0.2560	-0.2684
10	rs12563055	1	18895498	PAX7	0.2382	-0.0334
11	rs4659374	1	26651857	DHDDS	0.2352	-0.1673
12	rs2236815	1	18922533	PAX7	0.2322	-0.0385
13	rs766325	1	18829045	PAX7	0.2299	-0.0320
14	rs548436	3	174692475	NLGN1	0.2186	-0.1597
15	rs624761	1	18917464	PAX7	0.2145	-0.0849
16	rs553934	1	18885368	PAX7	0.2123	-0.0237
17	rs11576839	1	18876180	PAX7	0.2099	-0.0224
18	rs17261915	1	26629443	DHDDS	0.2093	-0.0773
19	rs7646820	3	174694326	NLGN1	0.2041	-0.0178
20	rs484341	3	174706113	NLGN1	0.1931	-0.2325
21	rs2237585	7	94887754	PON2	0.1865	-0.2865
22	rs585075	1	18893960	PAX7	0.1858	0.0462
23	rs753725	6	30998850	VARS2	0.1743	0.2634
24	rs2743208	1	18890442	PAX7	0.1685	-0.0230
25	rs2743189	1	18928969	PAX7	0.1658	-0.1081
26	rs485874	1	18945513	PAX7	0.1625	0.1000
27	rs42490	8	90847650	RIPK2	0.1351	-0.2038
28	rs2236817	1	18922202	PAX7	0.1266	0.0831
29	rs11247958	1	26639954	DHDDS	0.1264	-0.1338
30	rs618941	1	18878739	PAX7	0.1252	-0.0157
31	rs12760805	1	18894131	PAX7	0.1224	-0.1043
32	rs247970	3	174669421	NLGN1	0.1215	-0.1597
33	rs12437092	14	80074893	CEP128	0.1155	-0.1601
34	rs7633016	3	46703663	ALS2CL	0.1094	0.1824
35	rs4521967	1	18932426	PAX7	0.1089	0.0868
36	rs2596114	14	80198982	CEP128	0.1047	-0.1764
37	rs602875	6	32681607	HLA-DR–DQ	0.1042	-0.3019
38	rs9439731	1	18932058	PAX7	0.1008	0.0109
39	rs1868633	14	80078981	CEP128	0.0934	0.1250
40	rs16945848	15	60913837	TLN2	0.0926	0.3816
41	rs12121503	1	18947567	PAX7	0.0914	0.0125
42	rs2122252	14	80211444	CEP128	0.0913	0.4250
43	rs241448	6	32904662	TAP2	0.0911	0.1325
44	rs9270467	6	32666924	HLA-DRB1	0.0903	0.3776
45	rs9439725	1	18900904	PAX7	0.0891	0.0129
46	rs9270856	6	32678817	LOC100507709	0.0878	0.3776
47	rs4122190	6	31275831	HCG27	0.0873	-0.3076
48	rs11687301	2	38031029	FAM82A1	0.0844	-0.2231

49	rs7650366	3	46719970	TMIE	0.0833	0.1720
50	rs241409	6	32969898	LOC100294145	0.0821	0.3298
51	rs241451	6	32904458	TAP2	0.0800	0.2496
52	rs12817755	12	38585079	SLC2A13	0.0796	-0.3513
53	rs2236805	1	18930317	PAX7	0.0783	-0.0275
54	rs2074511	6	30997368	VARS2	0.0770	-0.1674
55	rs1343104	20	57607136	PHACTR3	0.0746	-0.1696
56	rs6728658	2	137556475	THSD7B	0.0724	0.1442
57	rs10502281	11	123261833	TMEM225	0.0714	-0.3682
58	rs3095319	6	31196016	PSORS1C1	0.0684	0.2124
59	rs2305100	13	43346934	CCDC122	0.0661	-0.3450
60	rs247979	3	174635453	NLGN1	0.0654	-0.0536
61	rs13396902	2	17781934	SMC6	0.0613	0.1347
62	rs12432505	14	80081792	CEP128	0.0594	-0.1589
63	rs1030809	3	174674737	NLGN1	0.0586	0.0207
64	rs447833	20	42696770	ADA	0.0578	0.2536
65	rs11632705	15	25141046	GABRG3	0.0570	-0.1925
66	rs2116538	2	137485890	THSD7B	0.0559	-0.1542
67	rs660962	11	106762375	CWF19L2	0.0559	-0.1205
68	rs2236816	1	18922341	PAX7	0.0558	0.0776
69	rs6092818	20	57741780	PHACTR3	0.0513	0.1507
70	rs17065164	13	43342706	CCDC122	0.0512	-0.3450
71	rs241444	6	32905087	TAP2	0.0508	0.1283
72	rs10507182	12	103729518	SLC41A2	0.0506	-0.1253
73	rs11900859	2	138039737	THSD7B	0.0505	0.1555
74	rs7350746	14	80122129	CEP128	0.0478	-0.0379
75	rs8019534	14	80239223	CEP128	0.0477	0.0007
76	rs9287459	2	137528489	THSD7B	0.0469	-0.1939
77	rs9783629	14	80220648	CEP128	0.0465	-0.1327
78	rs1469622	2	137592345	THSD7B	0.0465	-0.1088
79	rs1871457	14	80185504	CEP128	0.0459	-0.0863
80	rs1446296	2	38069920	FAM82A1	0.0456	-0.2380
81	rs2373327	2	38075590	FAM82A1	0.0453	-0.2531
82	rs241443	6	32905093	TAP2	0.0450	0.2528
83	rs1897419	2	1.37E+08	THSD7B	0.0449	0.1304
84	rs8003492	14	80155105	CEP128	0.0448	0.1024
85	rs10926272	1	2.39E+08	FMN2	0.0438	-0.0884
86	rs6544115	2	38006323	FAM82A1	0.0437	-0.1673
87	rs1805867	8	91100250	DECR1	0.0433	-0.1776
88	rs1265093	6	31215166	PSORS1C1	0.0431	-0.2017
89	rs2517598	6	30188253	TRIM31	0.0430	0.3718
90	rs2023472	6	30183843	TRIM31	0.0422	0.2222
91	rs10926178	1	2.38E+08	FMN2	0.0420	-0.2340
92	rs3819716	6	32912262	TAP2	0.0415	0.1278
93	rs6026998	20	57623030	PHACTR3	0.0409	0.1294
94	rs17110817	14	80120188	CEP128	0.0399	0.1517

Table S2. Additional simulation results under dominant genetic model.

Scenario	Average AUC		
	iBVS	LASSO	Stepwise
H70	0.902	0.885	0.866
H50	0.883	0.878	0.860
H30	0.784	0.771	0.765

Figure S1. Pairwise correlation coefficient R square of the 94 top SNPs

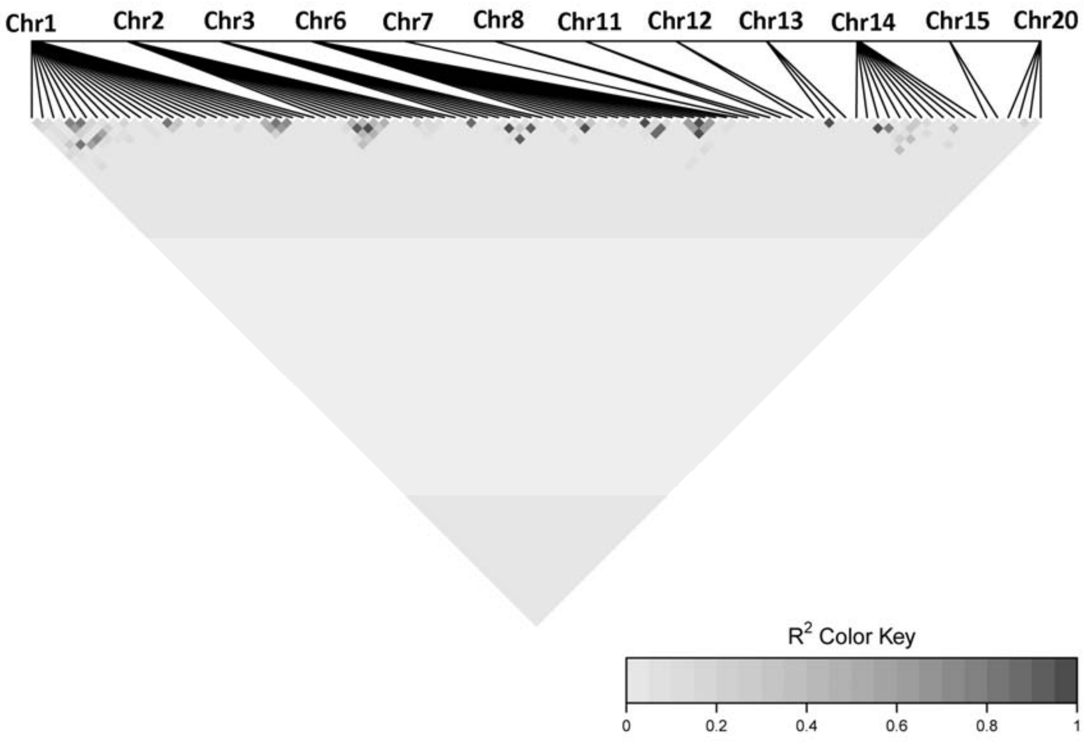

Figure S2. Posterior selection probabilities of SNPs under dominant genetic model in leprosy GWAS analysis.

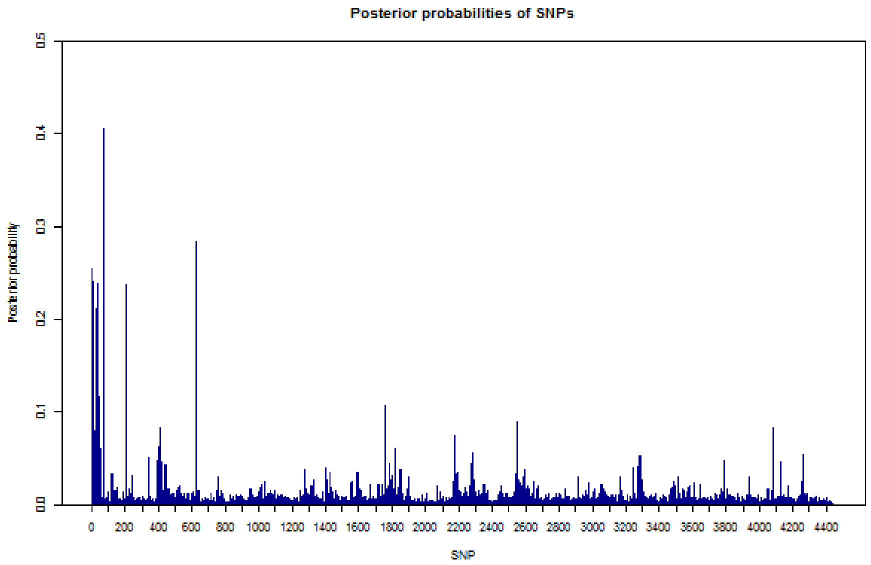

Constructing R Matrix Using KEGG

In the Application, we constructed the R matrix using KEGG (Kyoto Encyclopedia of Genes and Genomes) pathway, from which the gene interaction and reaction networks were extracted as our prior knowledge to be included in the model. We used Bioconductor packages "KEGG.db", "KEGGgraph" and "KEGGSOAP" to get the network information. The main steps with part of R codes are as follows:

(1) Find all the pathways IDs which include the genes in the Leprosy data.

```
number.gene <- length(geneID)
xx <- as.list(KEGGEXTID2PATHID)   # KEGGEXTID2PATHID maps Entrez Gene identifiers to KEGG
pathway identifiers.
pathID <- vector("list", number.gene)
for (i in 1:length(geneID))
{
   a <- as.numeric(geneID[i]==names(xx))
   b <- length(unique(a==0))
   if (b!=1)
   {pathID[i] <- xx[a==1] }
   else
   {pathID[i] <- NA}
}
```

(2) Download the above pathway from KEGG.

```
#Using getKGMLurl to download the pathway
getKGMLurl <- function (pathwayid, organism = "hsa",fpath="")
{
   baseurl <- "http://www.genome.jp/kegg-bin/download?entry="
   pathwayid <- gsub("path:", "", pathwayid)
```

```
  pco <- grep("^[a-z][a-z][a-z]", pathwayid)
  pco <- pco == seq(along = pathwayid)
  ispco <- length(pco) > 0 & all(pco)
  if (ispco) {
     organism <- sapply(pathwayid, function(x) substr(x, 1, 3))
     id <- pathwayid
  }
  else {
     id <- paste(organism, pathwayid, sep = "")
  }
  idfile <- paste(id, "&format=kgml", sep = "")
  urls <- paste(baseurl,idfile, sep = "")
  return(urls)
}

 pathid <- unique(unlist(pathID))
 for(i in 1:number.path)
 {
    idname <- pathid[i]
    fn <- paste("E:/…",idname,sep="")
    fn <- paste(fn,".xml",sep="")
    i
    download.file(getKGMLurl(pathwayid=idname,organism = ""),fn)
 }
```

(3) Merge all the pathways and conduct the R matrix by finding the neighborhood nodes.

```
xml.files <- list.files(path="E:/…",pattern=".xml")
nfile <- length(xml.files)
mapkegg <- lapply(xml.files,function(x){KEGGpathway2Graph(parseKGML(x))})
names(mapkegg) <- xml.files
merged.graphs <- mergeKEGGgraphs(mapkegg)    # mergeKEGGgraphs is used to merge graphs

nodes.graphs <- nodes(merged.graphs)
number.nodes <- length(nodes.graphs)
nodes <- translateGeneID2KEGGID(geneID, organism="hsa")

Rmatrix <- matrix(ncol=number.gene,nrow=number.gene,0)
for(i in 1:number.gene){
   nbor <- neighborhood(merged.graphs,nodes[i]) # neighborhood returns the neighborhood set of the given
node
   Rmatrix[i,] <- as.numeric(nodes %in% nbor[[1]])
}
```
